# Supplementary material for: From sequence to enzyme mechanism using multi-label machine learning
Source: BMC Bioinformatics. 2014 May 19;15:150. doi: 10.1186/1471-2105-15-150 (PMC4229970; doi:10.1186/1471-2105-15-150)
Supplement: Additional file 2 — Java code of ml2db. Additional file ml2db_code.tar.gz contains the Java source code to run the multi-label machine learning experiments and save the results to database. The code’s Javadoc is included. [file 1471-2105-15-150-S2.zip › additional file 2/ml2db/ml2db/doc/mulan/classifier/transformation/IncludeLabelsClassifier.html]

IncludeLabelsClassifier


JavaScript is disabled on your browser.


- Overview
- Package
- Class
- Use
- Tree
- Deprecated
- Index
- Help

- Prev Class
- Next Class

- Frames
- No Frames

- All Classes

- Summary:
- Nested |
- Field |
- Constr |
- Method

- Detail:
- Field |
- Constr |
- Method


mulan.classifier.transformation

## Class IncludeLabelsClassifier

- java.lang.Object
- - mulan.classifier.MultiLabelLearnerBase
  - - mulan.classifier.transformation.TransformationBasedMultiLabelLearner
    - - mulan.classifier.transformation.IncludeLabelsClassifier

- All Implemented Interfaces:
  :   java.io.Serializable, MultiLabelLearner, weka.core.TechnicalInformationHandler

  Direct Known Subclasses:
  :   AdaBoostMH

  ---

    

  ```
  public class IncludeLabelsClassifier
  extends TransformationBasedMultiLabelLearner
  ```

  A multilabel classifier based on the include labels transformation.
  The multiple label attributes are mapped to two attributes:
  a) one nominal attribute containing the class
  b) one binary attribute containing whether it is true.

  Version:
  :   2012.02.27

  Author:
  :   Robert Friberg, Grigorios Tsoumakas

  See Also:
  :   Serialized Form

- - ### Constructor Summary

    Constructors

    Constructor and Description

    `IncludeLabelsClassifier(weka.classifiers.Classifier classifier)`

    Constructor that initializes a new learner with the given base classifier
  - ### Method Summary

    Methods

    | Modifier and Type | Method and Description |
    | --- | --- |
    | `void` | `buildInternal(MultiLabelInstances mlData)` Learner specific implementation of building the model from `MultiLabelInstances` training data set. |

    - ### Methods inherited from class mulan.classifier.transformation.TransformationBasedMultiLabelLearner

      `getBaseClassifier, getTechnicalInformation, globalInfo`
    - ### Methods inherited from class mulan.classifier.MultiLabelLearnerBase

      `build, getDebug, isUpdatable, makeCopy, makePrediction, setDebug`
    - ### Methods inherited from class java.lang.Object

      `equals, getClass, hashCode, notify, notifyAll, toString, wait, wait, wait`

- - ### Constructor Detail


    - #### IncludeLabelsClassifier

      ```
      public IncludeLabelsClassifier(weka.classifiers.Classifier classifier)
      ```

      Constructor that initializes a new learner with the given base classifier

      Parameters:
      :   `classifier` -
  - ### Method Detail


    - #### buildInternal

      ```
      public void buildInternal(MultiLabelInstances mlData)
                         throws java.lang.Exception
      ```

      **Description copied from class: `MultiLabelLearnerBase`**

      Learner specific implementation of building the model from `MultiLabelInstances`
      training data set. This method is called from `MultiLabelLearnerBase.build(MultiLabelInstances)` method,
      where behavior common across all learners is applied.

      Parameters:
      :   `mlData` - the training data set

      Throws:
      :   `java.lang.Exception` - if learner model was not created successfully


- Overview
- Package
- Class
- Use
- Tree
- Deprecated
- Index
- Help

- Prev Class
- Next Class

- Frames
- No Frames

- All Classes

- Summary:
- Nested |
- Field |
- Constr |
- Method

- Detail:
- Field |
- Constr |
- Method
